# Supplementary material for: Comparative analysis of protein expression between oesophageal adenocarcinoma and normal adjacent tissue
Source: PLoS One. 2025 Mar 12;20(3):e0318572. doi: 10.1371/journal.pone.0318572 (PMC11902292; doi:10.1371/journal.pone.0318572)
Supplement: Attachment — Submitted filename: response-to-reviewers-2024-10-07.pdf [file pone.0318572.s005.pdf]

PONE-D-24-41870

Reviewer comments:

“We recognise that you obtained ethical approval from your local ethics committee after you began the study: your ethics approval document is dated 13 July 2018 while data were accessed on 30 April 2018. For human subjects research, PLOS ONE requires that authors obtain ethics committee approval before beginning the study, and we do not accept retrospective ethical approval.”

Response to reviewers:

The statement of an access date of 30th April 2018 was incorrect and an error on part, the correct date is 2nd October 2018. We apologise for this mistake and have updated the statement in the manuscript accordingly.
