# Supplementary material for: Comparative analysis of protein expression between oesophageal adenocarcinoma and normal adjacent tissue
Source: PLoS One. 2025 Mar 12;20(3):e0318572. doi: 10.1371/journal.pone.0318572 (PMC11902292; doi:10.1371/journal.pone.0318572)
Supplement: Attachment — Submitted filename: reviewer-comments-and-author-responses-2024-12-17.docx [file pone.0318572.s006.docx]

Reviewer comments and Author responses

We thank the editor and reviewers for their time and valuable comments. We are mindful that most aspects of academic publishing are unpaid and greatly appreciate the time and effort editing and reviewing takes. We have endeavoured to revise our manuscript accordingly.

## Academic Editor comments to the Author

### Academic Editor comment:

*Please revise the reviewers’ comments and modify the manuscript accordingly. In addition, I would like to highlight the need to include a sample size estimation showing how representative your sample is.*

### Author response

We agree that it is important to demonstrate the representativeness of samples. In this instance we know these are donors not an unbiased selection as they were all undergoing surgery, and whilst it is still possible to make a calculation with a small sample (here n=7), we feel it is more useful to make a direct comparison of the sample statistics to the underlying population statistics. In the UK population from which these samples were drawn, the most recent data for proportions of tumour stage at diagnosis were I = 5.8%, II = 11.4%, III = 24.4%, IV = 38% and unknown = 20.4%, and 69% of diagnosis are in men and 31% in women, with a median age of 71. Our samples were from tumour stages II = 28.6%, III = 57.1% and IV = 14.3% and our samples were all from men with a lower median age at diagnosis of 68. We feel that providing this comparison is more informative in terms of context and we have updated the abstract (Lines: 21-22) and introduction to include this information (Lines: 37-44). We have also clarified that these were a set of samples selected for tumour characterisation rather than population inferences.

### Academic Editor comment:

*Also, I suggest validating putative protein markers of oesophageal carcinoma. You can validate some markers by running a secondary analysis using external data, demonstrating their labeling by targeted methods such as western blot, or running an independent validation with additional samples.*

### Author response

We thank the Editor for this suggestion. This study was has demonstrated a large number of differentially expressed proteins in OAC, a number of which have been previously observed and validated. Where possible we have discussed our observations in the context of other studies, and the presence in our datasets of established biomarkers of OAC, combined with good separation by PCA, is reassuring. We agree that further validation would be ideal for the next phase of this work, and have the aim to extend the study to further validations in the future, should sufficient samples become available. The majority of previous data were gained by different methodologies or sample types than our own (e.g. Li et al,(2023) analysed proteins recovered from micro-dissected FFPE sections), and as such, can be challenging to directly compare with our data which were obtained from fresh frozen tumour and normal adjacent tissues,, thus we suggest that our data may provide a useful additional resource for biomarker studies in OAC.

## Reviewer 1 Comments to the Author:

### Reviewer comment

*Your PICO isn’t very clear in your abstract noe introduction, I would recommend clarifying it. I would recommend splitting your “Results” section to methods, analysis, and results instead of joining it all into one*

### Author response

We have revised the manuscript to reflect these suggestions both in structure and context. For the PICO we have revised Abstract (Lines: 21-30) and Introduction (Lines: 35-48).

## Reviewer 2 Comments to the Author:

### Reviewer comment

*Overall, this is an interesting observation study however the major limitations are first the sample size and second the lack of further analysis. The study would benefit from a more detailed focus in the discussion and avoid usign rather generic statements such as ‘suggesting that tumourigenesis may impact cellular phenotype’.*

### Author response

We have revised the manuscript to add some more detailed results and specific statements. Figure 1 has been revised to show the loadings arising from the proteins for PC1 and PC2. It indicates that the separation between tumour and normal adjacent tissue is primarily being driven by the common tumourigenesis markers keratin K1C18, AGR2 and antigen processing protein GILT. ( Lines: 57-63). The relative low expression of these 3 proteins for EN-430-11 can be seen in the heat-map of Figure 2B, and provides some explanation for the distance of OAC sample of EN-430-11 from the other OAC samples on the PCA plot. (Lines: 83-85). We have updated the discussion accordingly. (Lines: 109-121).

### Reviewer comment

*There are also quite a number of typographical errors - a few examples below: spelling- ‘mass spectrometery proteomics’ missing word and punctuation - For enrichment of ‘Reactome pathways [13] also found changes in’ capital letters- ‘Bligh EG, Dyer WJ. A RAPID METHOD OF TOTAL LIPID EXTRACTION AND PURIFICATION.’*

### Author response

Thank you for identifying these errors, we have endeavoured to fix them all during this round of revisions.

### Reviewer comment

*avoid discussing the results in the results section - for eg ‘Anterior Gradient 2 (AGR2) , involved protein folding and secretion was the most DEP for OAC. High AGR2 gene expression is a known unfavourable prognostic marker in renal and liver cancer [8].’*

### Author response

We have revised the results and discussion to address this issues. (Lines: 98-139).

### Reviewer comment

*avoid single sentence paragraphs eg ‘The principal value of the results presented here is the quantification of 3,500 proteins of which nearly 500 were differently expressed as a resource to other OAC researchers.’*

### Author response

We have revised the results and discussion to address this issues. (Lines: 108-122).

### Reviewer comment

*It is not clear if this refers to a previous study as the references do not correspond, or whether it was done during this study? ‘Additionally, the finding that the G95R mutation decreases the stability of NOP58 suggests…’*

### Author response

We apologise for this error and lack of clarity. The G95R mutation in NOP58 for donor EN-454-11 was previously identified in our previous paper DOI: 10.1111/imm.13578. The mass spectra is shown in Figure 3B in our previous paper. In the current manuscript, following the observation of lower expression of NOP58 for donor EN-454-11 we have calculated the effect of the G95R mutation on protein stability. We have revised the manuscript to make this clearer. (Lines: 86-92, 123-129).

## Reviewer 3 Comments to the Author:

### Reviewer comment

*The PCA shows that group PC1 contributes significantly higher variation. What is the nature of this group in component analysis?*

### Author response

We thank the reviewer for this question. We have revised Figure 1 to show the loadings arising from the proteins for PC1 and PC2. It indicates that the separation between tumour and normal adjacent tissue is primarily driving by common tumourigenesis markers keratin K1C18, AGR2 and antigen processing protein GILT. (Lines: 57-63).

The relative low expression of these 3 proteins for EN-430-11 can be seen in the heat-map of Figure 2B, and provides some explanation for the distance of OAC sample of EN-430-11 from the other OAC samples on the PCA plot. (Lines: 83-85)

### Reviewer comment

*The relative heterogeneity between the 7 patents seems quite obvious even in the DEP analysis. Does this heterogeneity stem from the small sample size or the personalized nature of each patient?*

### Author response

We assume the heterogeneity arises from both aspects and we have revised the manuscript to give more context for the samples with respect to the underlying population. Lines: Our revisions to the PCA plot for comparison with the heat-map aim to make clearer how these individual differences occur in the data, as noted in the previous response regarding the PCA and donor EN-430-11.

## Reviewer 4 Comments to the Author:

### Reviewer comment

*How did you identify G95R mutation decreases the stability of NOP58?How did you identify G95R mutation decreases the stability of NOP58?*

### Author response

The G95R mutation in NOP58 for donor EN-454-11 was previously identified in our previous paper DOI: 10.1111/imm.13578. The mass spectra is shown in Figure 3B in that reference. In the current manuscript, following the observation of lower expression of NOP58 for donor EN-454-11 we then used the DDGun tool to calculate the effect of the G95R mutation:

Montanucci L, Capriotti E, Frank Y, Ben-Tal N, Fariselli P. DDGun: an untrained method for the prediction of protein stability changes upon single and multiple point variations. BMC Bioinformatics [Internet]. 2019 Jul;20(S14). Available from: http://dx.doi.org/10.1186/s12859-019-2923-1

We have revised the manuscript to make this clearer (Lines: 86-92, 123-129) and the output table from DDGun is in the Supporting Information.

### Reviewer comment

*In this study, Is NOP58 differentially expressed that it gives a defective ribosomal product (DRiP)?*

### Author response

Our data cannot tell us that the NOP58 G95R mutation for donor EN-454-11 causes it to become a DRiP. However our data does show that NOP58 is has relatively lower expression for EN-454-11 and the G95R mutation is predicted to lower the stability of the protein, and we have our previous observation of a neoantigen peptide from the G95R region of NOP58. We speculate that collectively these three pieces of circumstantial evidence increases the likelihood that NOP58 is a DRiP for EN-454-11, but we cannot say with any certainty that this is so.

### Reviewer comment

*Apart from G2/M checkpoint related protein, CDC5L others genes/proteins are also differentially expressed, Why only these genes are targeted?*

### Author response

We apologise for the lack of clarity in the original manuscript submission, we have revised the results and discussion to include a larger subset of the differentially expressed proteins. (Lines: 57-63, 108-122).
