# Supplementary material for: Comparative analysis of protein expression between oesophageal adenocarcinoma and normal adjacent tissue
Source: PLoS One. 2025 Mar 12;20(3):e0318572. doi: 10.1371/journal.pone.0318572 (PMC11902292; doi:10.1371/journal.pone.0318572)
Supplement: Attachment — Submitted filename: reviewer-comments-and-author-responses-2025-01-08.docx [file pone.0318572.s007.docx]

Reviewer comments and Author responses

We thank the editor and reviewer again for their time and valuable comments. We have endeavoured to revise the manuscript accordingly.

## Reviewer 2 Comments to the Author:

*There are still some minor corrections that need to be made, for example:*

### Reviewer comment

*Notable in NAT were high expression in NAT of*

### Author response

We are unclear what revision the reviewer referring to as we could not identify the line with this text. The only line beginning with “Notable in NAT..” is Line 74:

“Notable in NAT were high expression of Protein-glutamine gamma-glutamyltransferase E (TGM3) and Heat shock protein beta-1 (HSPB1).”

### Reviewer comment

*be consistent when referring to companies and provide countries/headquarters for eg there is Waters, UK but no country etc for other companies.(Thermo Fisher Scientific,UK) but other times it is just Thermo Scientific*

### Author response

We thank the reviewer for identifying these oversights. We have corrected the Materials and Methods accordingly (Lines 159-181).

### Reviewer comment

*use lowercase n for ‘specifically Neutrophil degranulation’*

### Author response

We have corrected the case (Lines 97 and 136).

### Reviewer comment

*I think there should be a bit more emphasis on studies that have previously identified the same proteins eg for AGR in Barretts https://pmc.ncbi.nlm.nih.gov/articles/PMC2575112/.*

### Author response

We have revised the discussion to include more evidence for the role of AGR2 in tumourigenesis (Lines 114-117).

“Moreover, the role of AGR2 in tumourigenesis in the oesophagus has previously been seen in higher gene expression of AGR2 in OAC precursor condition Barrett’s oesophagus with respect to NAT (Hao, 2006), and increased expression of AGR2 in fibroblast cells was seen to promote tumour growth in mice (Wang, 2008).”
